# Pointers to Interventions for Promoting COVID-19 Protective Measures in Tourism: A Modelling Approach Using Domain-Specific Risk-Taking Scale, Theory of Planned Behaviour, and Health Belief Model

**DOI:** 10.3389/fpsyg.2022.940090

**Published:** 2022-06-29

**Authors:** Timo Ohnmacht, Andreas Philippe Hüsser, Vu Thi Thao

**Affiliations:** Institute of Tourism and Mobility ITM, Lucerne University of Applied Sciences and Arts, Lucerne, Switzerland

**Keywords:** Theory of Planned Behaviour (TPB), Health Belief Model (HBM), risk taking measurement, intervention design, tourism, COVID-19

## Abstract

Based on the factors of the Theory of Planned Behaviour (TPB), the Health Belief Model (HBM), and the DOSPERT scale, used to measure general risk-taking behaviour, a combined model has been developed for investigating tourists’ intentions to implement protective measures against the coronavirus disease 2019 (COVID-19). The purpose of the study is to formulate a model that Swiss tourism practitioners can use to understand tourists’ decision-making regarding the acceptance and proper implementation of non-pharmaceutical interventions (NPIs). A large-scale cross-sectional population study that is representative for the Swiss population has been designed to validate the model (*N* = 1,683; 39% response rate). In our empirical investigation, a simple regression analysis is used to detect significant factors and their strength. Our empirical findings show that the significant effects can be ordered regarding descending effect size from severity (HBM), attitude (TPB), perceived behavioural control (TPB), subjective norm (TPB), self-efficacy (HBM), and perceived barriers (HBM) to susceptibility (HBM). Based on this information, intervention strategies and corresponding protective measures were linked to the social-psychological factors based on an expert workshop. Low-cost interventions for tourists (less time, less money, and more comfort), such as the free provision of accessories (free mask and sanitizers) or free testing (at cable cars), can increase the perceived behavioural control and lower the perceived barriers and thus increase the acceptance of this protective measure.

## Introduction

Since the outbreak of the coronavirus disease in 2019 (COVID-19), tourists need to apply various protective measures as infection risk-reducting practises before, during, and after travelling. Protective measures include non-pharmaceutical interventions (NPIs), such as disinfecting one’s hands, social distancing, health screening and testing, coughing and sneezing etiquette, the correct wearing of masks, going into quarantine upon arrival, or when returning from abroad and travel bans (WHO, 2021). These NPIs are combined with pharmaceutical interventions (PIs), such as proof of two doses of vaccine plus one or more boosters. This variety and combination of (N)PIs has affected and changed people’s decision-making in the domain of tourism based on their perceptions of risk (see Williams and Baláž, 2013, 2015; Yang and Nair, 2014 for discussions on tourism and risk perception).

A prerequisite for safe travel is that tourists implement specific protective measures into their tourism practises. Bratić et al. (2021, p. 10) state that the revival of tourism requires “behaviour specific strategies to reduce behaviour specific travel anxiety.” As a consequence, the pandemic has increased the need for research on intervention designs to promote COVID-19 protective measures in tourism (WHO, 2021). In particular, the massive reduction in tourist arrivals worldwide (UNWTO, 2021), including in Switzerland (Georgi et al., 2020; STV, 2020; TCS, 2021), due to the pandemic has given a tremendous boost to tourism research focusing on tourists’ decision-making and perceptions of risk (e.g., Abraham et al., 2020; Bae and Chang, 2020; Agyeiwaah et al., 2021; Bratić et al., 2021; Golets et al., 2021; Neuburger and Egger, 2021; Pappas, 2021).

In research in social psychology, popular theories and methods have been developed to identify the factors that influence the intentions and consequently the behaviour patterns that have been adopted into the domain of tourism (Li et al., 2021; Rather, 2021a,b; Han et al., 2022). This new stream of literature builds on the assumption that COVID-19 affects travellers’ deliberative decision-making about their travel intentions. This includes tourists’ intention to implement interventions (correctly) whilst travelling to enhance the physical health of both themselves and others (e.g., Das and Tiwari, 2021). The combination of different theories in psychological research on health-related behaviour is very promising in enabling a better understanding of health-related behavioural changes (Champion and Skinner, 2008; Aiken, 2011; see also Huang et al., 2020).

Against this background, our study context is 3-fold: (1) to develop a model that is of practical use for tourism practitioners that captures a wide range of mutually exclusive socio-psychological influencing factors affecting tourists’ intentions to apply to protective measures against COVID-19; (2) to test this model empirically in order to detect significant factors and their strength; and subsequently (3) to formulate pointers to interventions based on significant factors increasing the intention to apply (N)PIs in order to enhance safe travel during pandemics.

Our approach focuses on the deliberative decision-making processes which, in socio-psychological research, are prominently captured by the Theory of Planned Behaviour (TPB; Ajzen, 1991). Within a pandemic, those decision-making processes can best be extended by the factors of the Health Belief Model (HBM; Rosenstock, 1966; Champion and Skinner, 2008). From a practical point of view, the advantages of combining these two theories lies in developing pointers to interventions based on the outputs of the statistical modelling. The results can be used to evaluate interventions that address the question of socio-psychological influences. The selection of the components of the theories, have advantages for the policy dimension and discussions of the design of interventions (Aiken, 2011). Based on our study context, we have developed a theory-based explanatory model by building on the TPB, combining it with a HBM, and adding the DOSPERT risk-taking scales in the domains of recreation and tourism to determine the general risk dispositions of tourists (DOSPERT; Weber et al., 2002; Blais and Weber, 2006).

The remainder of this paper is structured as follows. First, current theories on perceptions of risk are discussed in the form of a literature review, and empirical findings in our study field are presented. Second, we present a combined explanatory model from a theoretical perspective. Third, to provide evidence for our theoretical model, our own empirical findings from a representative population study in Switzerland during the coronavirus pandemic are presented and discussed. The paper will conclude with a presentation of exemplary protective measures that can be linked theoretically to the significant influencing factors within our model. This serves as a starting point for evidence-based intervention research on safe travel that can guide further research. This research is supported by the Swiss National Science Foundation (SNSF) within the framework of the National Research Programme “COVID-19” (NRP 78) Grant-N° 4078P0_198336.

## Materials and Methods

### Expected Utility Theory vs. the Psychometric Paradigm

The early literature on risk distinguishes risk with *known* uncertainties (known risk) from risk with *unknown* uncertainties (unknown risk) (Knight, 1921; Williams and Baláž, 2013, 2015). For example, behavioural economists and social scientists have defined risks in the area of gambling in an objective manner, where individuals are supposed to make risky choices between different monetary outcomes with known probabilities (e.g., Kahneman and Tversky, 1979, 1984). In this tradition, individuals’ risk preferences for lotteries are derived from the expected utility framework (e.g., Machina, 1982) and classified according to whether the shape of the individual utility function is risk-averse or risk-seeking (Kahneman and Tversky, 1979, 1984; Lopes, 1987; Levy, 1992). Conversely, when the probabilities of outcomes are unknown or when people lack any evidence about the underlying probabilities, this is called a decision or judgement under *uncertainty* (Tversky and Kahneman, 1974; Lopes, 1987; Williams and Baláž, 2013, 2015). Research by experimental psychologists has shown that people rely on heuristic cues when making judgements about uncertain outcomes, which can lead to severe biases (Tversky and Kahneman, 1974; Gilovich et al., 2002; for a review of the cognitive biases in tourists’ decision-making, see Wattanacharoensil and La-ornual, 2019).

Contrary to the expected utility in the domain of rational-choice theory is the risk model of Slovic and his colleagues, which is based on the so called “psychometric paradigm” (Slovic et al., 1984; Slovic, 1987, 1992; for an overview of psychometric theory, see Raykov and Marcoulides, 2011). According to stream of research, risk is not objective but depends upon individual judgements. That is, risk judgements are subjective and based on intuitions, which are commonly referred to as “risk perceptions” (Slovic, 1987, p. 280, 1992). This stream of research has investigated the factors underlying risk perceptions and risk attitudes based on individual judgements evaluated on rating scales, and has employed multivariate statistical analyses such as principal components and multidimensional scaling (Fischhoff et al., 1978; Slovic et al., 1984; Slovic, 1987, 1992; Siegrist et al., 2005).

### Tourism and Risk Perception

Most of the literature on tourism risks has focused on perceived (subjective) risks (Rather, 2021a,b; Han et al., 2022), rather than objective risks that are known, given that tourism mostly involves uncertainties that cannot be quantified with objective probabilities (Williams and Baláž, 2013; for a review of risks and uncertainties in tourism, see Yang and Nair, 2014; Williams and Baláž, 2015). In tourism research, risk has been defined on the micro-level as the *perceptions* and *experiences* of individual tourists about the conditions and potentially negative consequences of touristic services or products whilst purchasing tourism and travel, as well as at the tourist destination (Tsaur et al., 1997; Reisinger and Mavondo, 2005). Many researchers have focused on how risk perceptions influence decision-making and travel behaviour (e.g., Reisinger and Mavondo, 2005; Kozak et al., 2007; Rittichainuwat and Chakraborty, 2009; Neuburger and Egger, 2021), whilst other researchers have concentrated on the dimensions determining tourists’ risk perceptions (e.g., Fuchs and Reichel, 2006; Reichel et al., 2007; Jonas et al., 2011).

There is vast literature on how individual tourists’ risk perceptions associated with COVID-19 affect their travel intentions and behaviour (e.g., Abraham et al., 2020; Agyeiwaah et al., 2021; Chua et al., 2021; Golets et al., 2021; Kim et al., 2021; Meng et al., 2021; Neuburger and Egger, 2021; Rather, 2021a,b; O’Connor and Assaker, 2022; Wu and Lau, 2022). A few authors have applied the Theory of Planned Behaviour (TPB) to capture risk perceptions of the COVID-19 situation and their impact on tourists’ intentional behavioural outcomes (Bae and Chang, 2020; Li et al., 2021; Liu et al., 2021; Sánchez-Cañizares et al., 2021; Seong and Hong, 2021; Sujood and Bano, 2021). This stream of literature has in common the fact that the authors extend the three standard explanatory constructs of attitude, subjective norms, and perceived behavioural control to predict (travel) intentions by adding other explanatory dimensions in order to capture perceptions of risk. These approaches are postulated either as antecedents or as tantamount to the standard three explanatory constructs, and they result in an “extended Theory of Planned Behaviour (eTPB)” (Bae and Chang, 2020, p. 1018).

Based on this research stream, there is ample evidence that COVID-19 strongly influences perceptions of health and travel risks that themselves influence intentions regarding travel behaviour. For example, besides the three TPB elements, Bae and Chang (2020) included in their survey the two latent constructs of cognitive and affective risk perceptions to explain behavioural intentions. Their notion of cognitive risk perception is taken from the Health Belief Model (HBM; Rosenstock, 1966; Champion and Skinner, 2008), which is attached in separate parts to the TPB. In particular, the HBM explanatory factor of “susceptibility” is commonly used for eTPB. Findings of Bae and Chang (2020) provide nuanced insights into the leading influencing factors, most importantly that susceptibility has a comparatively important positive impact, extending the classical three influence dimensions of TPB to the behavioural intention to practise what they call “untact” tourism (a Korean term for minimising contact between tourists).

However, alongside these exemplary studies focusing on risk perceptions and travel intentions in general, fewer studies in tourism research concentrate on the intention to implement interventions against COVID-19 before and whilst travelling within the eTBP approach. For example, before COVID-19, Lee et al. (2012) extended the model of goal-directed behaviour (MGB), which is likewise an extension of TPB, with information on past behaviour in the case of the influence of H1N1. They found that perceptions of the severity of 2009 H1N1 had a positive effect on the acceptance of NPIs whilst travelling. A more recent study by Das and Tiwari (2021) investigated the intention of international and domestic tourists in India to adopt NPI differentiated by sociodemographic characteristics based on an “unrestricted, self-selected survey” (p. 232). This study can be seen as one of the first approaches to investigate how perceptions of the severity of COVID-19 influence the intention to adopt NPIs. Importantly, they can show that the perceived severity of COVID-19 is indirectly associated with travel intentions through an intention to adopt NPIs. This is consistent with the findings of Liu et al. (2021), who likewise found that the impact of the perception of COVID-19 on post-pandemic travel intentions was partially mediated through adherence to NPIs.

Based on our reading of the literature, we conclude that, thus far, tourism researchers have attempted to define perceptions of risk by applying additional constructs that extend the TPB and comparable theories. This approach can be linked to the “psychometric paradigm” and the subjective risk perception model of Slovic (1987).

Whilst most papers focus on travel avoidance and intention (Cahyanto et al., 2016; Abraham et al., 2020; Agyeiwaah et al., 2021), only a few studies have put intentions to implement interventions against COVID-19 whilst travelling at the centre of their research (Lee et al., 2012; Das and Tiwari, 2021; Liu et al., 2021). A further drawback of the literature is that the dynamic situation of the pandemic has led to somewhat fast data-gathering procedures. This is obvious because a lot of studies rely on non-probabilistic snowballing samples or the random recruitment of study participants from the internet (e.g., social network platforms; Golets et al., 2021; Pappas, 2021). Additionally, whilst most researchers can detect the factors that influence their research and discuss them with regard to the implications for management, they do not discuss them with tourism experts in more detail with a view to formulating recommendations for the design of interventions.

Based on these shortcomings, we argue that there is a need for a holistic model that is likewise applicable for the practitioners that incorporates risk perceptions into research on travellers’ intentions to implement NPIs.

### A Combined Model

In the interests of broadening theory, we propose that risks in the sector-specific domain of tourism can best be captured by combining models from social psychology, health research, and domain-specific theories of risk regarding recreation and tourism. The TPB has mostly been applied for pro-environmental behaviour (Gholamrezai et al., 2021). The Health Belief Model was used, e.g., for the improvement of oral health behaviour (Sanaeinasab et al., 2022), condom use (Montanaro and Bryan, 2014), helmet use (Quine et al., 1998), and preventive behaviour analysis against sunlight (Moradhaseli et al., 2019). A combination of TPB and HBM is used as a research framework for rural dwellers’ intention to adopt sustainable water management (Aliabadi, 2020).

The previously discussed gap in tourism research will be addressed through the formulation of a combined model that incorporates the TPB (Ajzen, 1991), the HBM (Rosenstock, 1966; Champion and Skinner, 2008), and the Domain-Specific Risk-Taking scale (DOSPERT; Weber et al., 2002; Blais and Weber, 2006).

We have seen in our literature review that application of the TPB (Ajzen, 1991) is well established in tourism research as a way of predicting people’s intention to travel. However, under the new circumstances of a pandemic, this broadly used general theory must be adjusted in the direction of assessing tourists’ perceptions of risk. Ajzen (2020, p. 317) takes the position that, for reasons of sufficiency and parsimony, no additional constructs should be added to the three explanatory dimensions (attitude, subjective norms, and perceived behavioural control) in explaining intentions. However, he further states that in principle the TPB is open to “the inclusion of additional predictors” and that these “additions should be conceptually independent [and thus mutually exclusive and statistically independent; the authors] of the theory’s existing predictors, rather than be redundant with them” (Ajzen, 2020, p. 317).

According to Ajzen, the antecedent variables that explain the three factors of the TPB are behaviour, norms, and control beliefs. From our perspective, the perception of risk can be treated as an independent explanatory dimension whose relevance has been increased by the COVID-19 pandemic; it should thus be included in the combined explanatory models. Since perceptions of risk can be theory-based and understood as the deliberative assessment of perceived outcomes, we see these factors as equivalent to the three explanatory dimensions of TPB. In this case, the common method variance approach should be assessed especially for cross-sectional data (Podsakoff et al., 2003; Rasoolimanesh et al., 2021; Rather et al., 2021).

#### A Combined-Theory-Based Explanatory Model for Predicting the Intention to Implement Interventions Against COVID-19 Whilst Travelling

**Figure 1:**
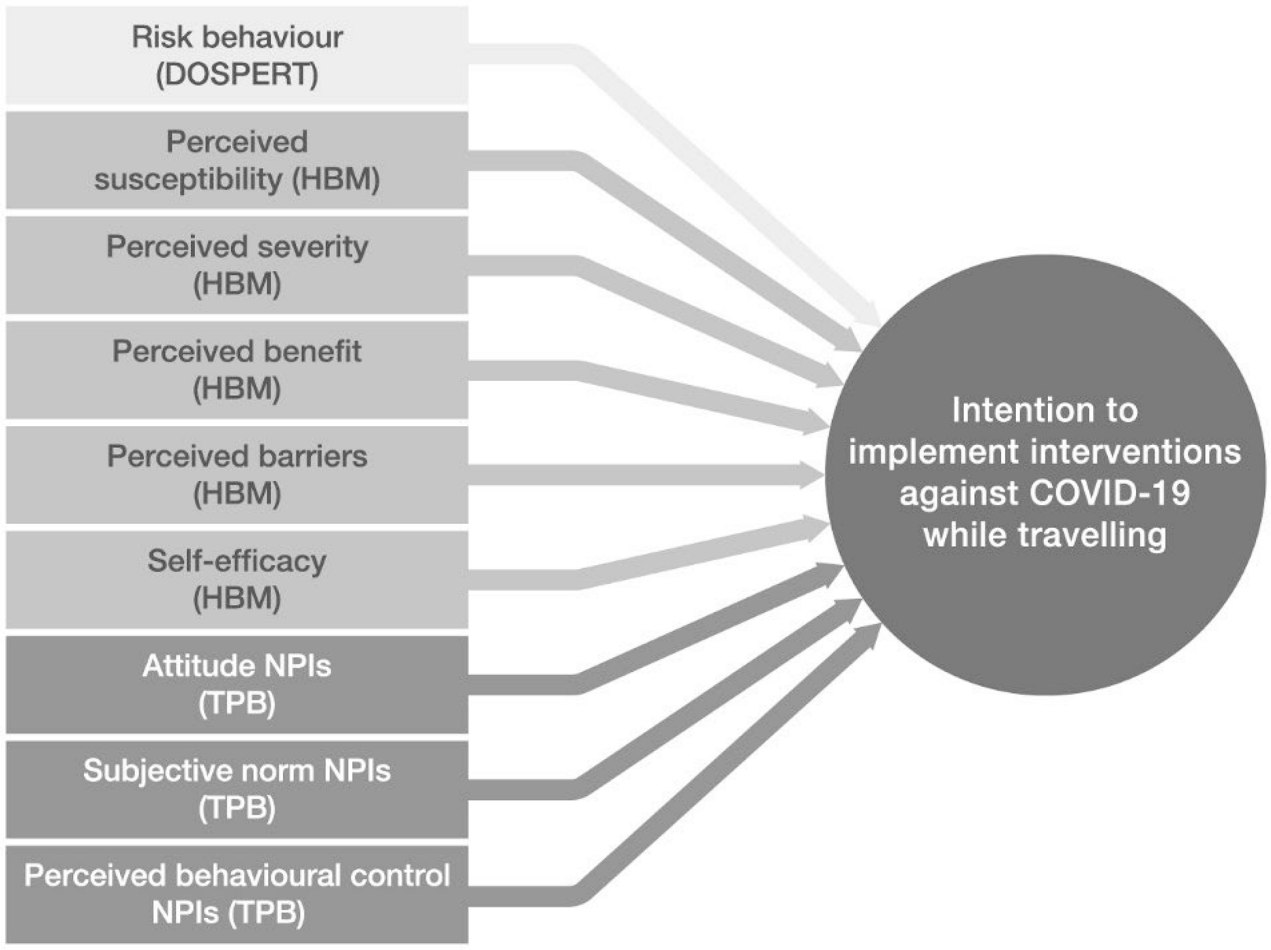
The combined explanatory model, with intention to implement non-pharmaceutical interventions [(N)PIs] as a dependent variable.

The present explanatory model for predicting the intention to travel under the threatening situation of a pandemic combines the *TPB*, *HBM*, and *DOSPERT*. The combination of two social-psychological explanatory models (TPB and HBM), both attitude-behaviour models (Hüsser, 2016), with the scale for measuring risk behaviour for recreation (*DOSPERT*) provides a theoretical foundation for the development of evidence-based and suitable evaluations of protective measures for safe travel. Figure 1 shows the combined model.

#### Psychometric Scales for Determining Influences Explaining the Intention to Implement Interventions Against COVID-19

The following psychometric scale (DOSPERT) and two explanatory models (TPB/HBM) not only focus on determining cognition with respect to dangers and threats (risk perception), but they are also used to explain and predict behavioural intentions. Within this application, the model is used to improve explanation and prediction of the intention to implement interventions against COVID-19 whilst travelling.

##### The Domain-Specific Risk-Taking Scale

The Domain-Specific Risk-Taking Scale (DOSPERT) is a psychometric scale for measuring risk perceptions and behaviours (see Weber et al., 2002). This scale for measuring the risk-taking attitudes of individuals has been developed and validated for different domains (Weber et al., 2002; Johnson et al., 2004; Blais and Weber, 2006). Based on rating scales, respondents must assess their probability of pursuing a certain activity or engaging in certain behaviour. In tourism research, the DOSPERT scale has been found to predict risky health behaviour amongst Swiss travellers to Thailand (Farnham et al., 2018). In the context of adherence to COVID-19 protective measures, the higher risk-tendency scores of the DOSPERT scale were significantly correlated with more active corona risk behaviour (Keinan et al., 2021). Consistent with this finding are the results of Guenther et al. (2021), who were able to show that higher risk-propensity scores in the DOSPERT scale were negatively correlated with precautions associated with risky COVID-19 behaviour. Another study by Fini et al. (2021) showed that, when it comes to social distancing, the preferred interpersonal distance depends on perceptions of health and safety risks as measured by the DOSPERT scale.

In the present research, the DOSPERT scale was chosen because of its satisfactory reliability, its validation across different domains, and its availability in different languages (e.g., Farnham et al., 2018; Shou and Olney, 2020).

##### The Health Belief Model

The Health Belief Model (HBM; Rosenstock, 1960, 1966, 1974; see also Champion and Skinner, 2008) was initially developed to predict individuals’ health behaviour in preventing diseases. There are five key constructs that predict whether an individual will take action to avoid an undesirable health condition. The first construct is that of perceived susceptibility, which refers to an individual’s beliefs about the chances of being at risk of contracting a disease. The second construct is that of the perceived severity of contracting a disease. Perceived severity includes both beliefs about possible medical consequences, such as physical or mental impairments, and the broader social consequences, for example, adverse effects on work, social relations or the family. Perceived benefits are beliefs about the perceived efficacy of a behavioural change or available actions for the purpose of reducing the risk of threat and the severity of an undesirable health condition. The fourth construct, perceived barriers, are a person’s beliefs about the perceived impediments of taking a recommended action, that is, an action or procedure that might be “inconvenient, painful, expensive, time consuming, and difficult to perform” (Rosenstock, 1960, p. 296). Self-efficacy, the last construct, refers to a person’s confidence and expectations in his or her own capabilities to perform an action or form of behaviour or change in behaviour that leads to the desired result. The concept of self-efficacy was initially introduced by Bandura (1977). Rosenstock et al. (1988) proposed to incorporate self-efficacy as an additional independent variable into the Health Belief Model. Self-efficacy is an important prerequisite in health behaviour, given that (strong) self-efficacy can initiate and maintain changes to health behaviour (Bandura, 1977; Strecher et al., 1986; Rosenstock et al., 1988).

In tourism research, for example, the Health Belief Model was employed to predict travel avoidance due to Ebola in the United States (Cahyanto et al., 2016). More precisely, Cahyanto et al. (2016) found that perceived travel risks and perceived susceptibility were both associated with a higher probability of travel avoidance, whereas self-efficacy was associated with lower probabilities of travel avoidance. However, they found no significant effect of perceived severity. Another study by Huang et al. (2020) combined the Health Belief Model with the Theory of Planned Behaviour using a structural equation model approach. Likewise, the results indicated that self-efficacy, perceived susceptibility, and perceived benefits, but not perceived severity, had a positive direct effect on preventive health behaviour. Moreover, the effect of perceived susceptibility and perceived benefits exerted an indirect effect on preventive behaviour through attitudes towards the preventive behaviour (mediation).

##### The Theory of Planned Behaviour

The Theory of Planned Behaviour (TBP; Ajzen, 1985, 1991; Ajzen and Fishbein, 2005) states that behavioural intention is the main antecedent of the behaviour of individuals if the behaviour is under volitional control. Behavioural intention comprises the motivational components to perform a behaviour. The higher the intention and therefore the motivation to perform the behaviour, the more likely it becomes that people engage in it. The behavioural intention itself comprises three determinants. The first determinant is one’s personal attitude towards the behaviour, which refers to peoples’ positive or negative evaluations of the behaviour under consideration. The second determinant is the subjective norm, or one’s perceptions of whether people who are important and close to you think the behaviour should be performed or not. Finally, perceived behavioural control refers to “people’s perception of the ease or difficulty of performing the behaviour of interest” (Ajzen, 1991, p. 188). That is, people must not only be motivated but also have the ability and skills to carry out the behaviour, such as time and information. The more favourable a person’s attitude towards the behaviour, the more favourable the subjective norm, the higher the perceived behavioural control, and the higher the intention to perform the behaviour.

#### Presentation of the 10 Constructs

All 10 constructs of the combined model are presented in Table 1.

**Table 1:** The 10 constructs of the combined model.

	Explanation of construct
DOSPERT	
Risk behaviour (in recreation and sports)	A person’s intention to take risks in their leisure time
HBM	
Perceived susceptibility	Individuals’ assessments regarding the risk of coronavirus infection whilst travelling
Perceived severity	Individuals’ assessments regarding the severity and consequences of a possible infection with the coronavirus
Perceived benefit	Individuals’ assessments regarding the benefits of protective measures against the coronavirus when travelling
Perceived barriers	Individuals’ assessments regarding the drawbacks of protective measures against the coronavirus when travelling
Self-efficacy	Individuals’ assessments regarding the extent to which they can contribute to the ending of the pandemic with their own behaviour
TPB	
Attitude	Respondents’ attitudes towards implementing interventions during touristic travel
Subjective norm	Influence of people who are important to the respondent regarding implementing interventions during touristic travel
Perceived behavioural control	Availability of the necessary resources regarding implementing interventions during touristic travel
Intention	Intention to implement interventions against COVID-19 during touristic travel

Own presentation.

#### Hypothesis for the Combined Model

The aim of this research is firstly, to confirm the methodological and statistical possibility of the combination of the three-explanation dimension (TPB, HBM, and DOSPERT); secondly, to identify, and thirdly, to rank, compare, and contrast the significant effect sizes of the various influencing factors (constructs). Taking into consideration previous research by Lee et al. (2012), Cahyanto et al. (2016), Bae and Chang (2020), Das and Tiwari (2021), and Liu et al. (2021) justify and guide the formulation of our hypothesis regarding TPB and HBM (direction of the effects).

Our research proposes three levels of hypotheses. The first level is related to the combination of the three explanatory models into one model. Firstly, the question arises whether the five HBM and the four TPB factors in the combined model can all be considered conceptually independent. In statistical terms, this combination does not produce multi-collinearity and thus redundancy between the model’s explanatory factors, and there is no common method bias (Fuller et al., 2016). Therefore, the following working hypotheses are proposed:

*H1.1a*: The Variance Inflation Factor (VIF) for each of the nine factors does not exceed 10.

*H1.1b*: There is no common method bias based on Harman’s single factor test.

Secondly, we want to examine whether the combined model we propose has greater explanatory power than the models taken individually, that is:

*H1.2*: The explained variation of the combined model exceeds the explained variation of the component models.

*H1.3*: Due to the combination of the explanatory factors in one model, the causation of the factors (*ceteris paribus*) can be controlled in order to produce a better assessment of the effect sizes.

The second level is related to the direction and significance of the influencing factors. Overall, these hypotheses can be separated into the three parts of the explanatory models:

##### Domain-Specific Risk-Taking Scale

*H2.1*: The greater the general level of risk-taking behaviour by tourists, the lower the intention to implement interventions against COVID-19.

##### Health Belief Model

*H2.2*: The greater the perceived susceptibility to the coronavirus when travelling, the greater the intention to implement interventions against COVID-19.

*H2.3*: The greater the perceived severity of the possible progression of an illness, the greater the intention to implement interventions against COVID-19.

*H2.4*: The greater the perceived benefit of protective measures against the coronavirus when travelling, the greater the intention to implement interventions against COVID-19.

*H2.5*: The greater the perceived barriers in implementing protective measures against the coronavirus when travelling, the lower the intention to implement interventions against COVID-19.

*H2.6*: The greater the perceived self-efficacy regarding one’s behaviour and protective measures against COVID-19, the greater the intention to implement interventions against COVID-19.

##### Theory of Planned Behaviour Non-pharmaceutical Interventions

*H2.7*: The more positive the attitude towards (N)PIs, the greater the intention to implement interventions against COVID-19.

*H2.8*: The more positive the subjective norm towards (N)PIs, the greater the intention to implement interventions against COVID-19.

*H2.9*: The greater the perceived behavioural control, the greater the intention to implement interventions against COVID-19.

The third level addresses the ranking of significant factors according to the size of their effect. It is argued that knowledge about the leverage effect of each influencing dimension forms a basis for discussion of the effectiveness of pointers of interventions and safety measures from a policy perspective.

*H3*: Perceived susceptibility and perceived severity can both be seen in terms of effect size as the leading factors of the model.

Based on our literature review, Das and Tiwari (2021, p. 237) showed that females and the elderly have a greater intention to adopt safety measures. Furthermore, because further research has found that older tourists perceive COVID-19 as more severe and likewise show a greater acceptance in applying interventions, we state an additional hypothesis on the interaction between age and severity for our combined model (*ibid.*, p. 235). That is, we hypothesise that the effect of severity on (N)PIs adherence whilst travelling is more pronounced amongst the elderly than amongst the young.

*H4*: Females have greater intentions to implement interventions against COVID-19.

*H5*: The older the tourist, the greater intention to implement interventions against COVID-19.

*H6*: There is a two-fold effect in the interaction between age and severity on the intention to implement interventions against COVID-19, the intention being stronger amongst the elderly than amongst the young.

### Methods

Our empirical research is based on a survey that is representative of the Swiss population aged 18 and above. It is designed firstly, to confirm the possibility of generating a theory-based combined explanatory model (i.e., no multi-collinearity between the explanatory factors and a higher explanatory power of model variations); and secondly, to discuss significant effects and their effect sizes regarding their usefulness in designing the intervention.

#### Sampling and Procedure

In order to investigate the postulated model (see Figure 1), a trilingual (German, French, and Italian) and cross-sectional national survey of the Swiss resident population aged 18 and above was carried out between March and May 2021. A letter of invitation to participate in the study was sent by post to a total of 4,530 randomly selected persons residing in Switzerland. The envelope included a pen and paper questionnaire and a prepaid return envelope. It was also possible to fill out the survey online *via* a QR link on the invitation letter or by typing the URL into a web browser. Field support was provided by members of the research team based on a support email address and a telephone hotline. After 3 weeks, a wave of reminder communications was initiated for those who had not yet answered the survey, including all printed material from the first wave. Our sample population was taken from CASTEM [*Cadre de Sondage pour le Tirage d’Echantillons de Ménages* (Sampling frame for drawing household samples from the census)]. This sampling frame was provided by the Swiss Federal Statistical Office based on the federal population census (FSO, 2021). Table 2 shows the total of 4,530 people contacted, of whom 164 were reported as unreachable. A total of 1,683 persons participated in the survey. This corresponds to a comparatively high response rate of 39%. The data are available on an open science repository (Ohnmacht and Hüsser, 2022).

**Table 2:** Analysis of the sample response rate.

	*n*	%
Gross sample	4,530	100
Non-sampling relevant losses (moved, deceased, wrong address, etc.)	164	4
Net *sample*	4,366	100
Response online	390	9
Response by pen and paper	1,293	30
Response total	1,683	39

Own survey data.

#### Operationalization and Validation of the Constructs

The dynamic situation of the COVID-19 pandemic concerning the development of safety measures and the enforcement of implementation needs careful operationalization, especially of the outcome variable. Ajzen (2020) suggests that behavioural intentions should be linked to target, action, context, and time (TACT). The TACT of our dependent variable, “intention to implement interventions against COVID-19,” consists of the target “holiday trips,” excluding business travel; action is addressed with the application of protective measures against COVID-19; context is framed such that adherence is voluntary; and time is referred to with regard to the “next trip,” which was framed as “holiday-making in the year 2021.”

In our operationalization of the outcome variable, we asked respondents to imagine that applying protective measures is entirely voluntary. From our perspective, this form of operationalization is needed first, due to the dynamic nature of the situation, which can be understood from an environmental socio-psychological perspective as an environmental context (political, social, and cultural). For discussions of environmental contexts, see Ohnmacht et al. (2017), and for the connection between degrees of freedom and travel-decision modelling, see Laesser et al. (2018, p. 621), who state that “[o]pportunity signifies the availability of exogenously favourable/supportive conditions that enable an action.” Second, measuring the intention with signs of voluntariness serves as a better starting point for the intervention design from the perspective of behavioural change. Third, if the interventions are framed as mandatory, methodologically speaking, ceiling effects appear for the scale measures that restrict the modelling of the effects.

The survey design covers the following sections with regard to the operationalization of the constructs: reflective indicator measurements (three to eight items per latent variable) and socio-demographic information. All constructs shown in Figure 1 were collected based on a multitude of items on reflective measurement scales. The individual assessments of the survey items were measured using five-point Likert scales. The endpoints were named, but not the gradations, in order to treat the scale points as equidistant for purposes of quasi-metric analysis. The items were taken from the relevant literature (e.g., Quine et al., 1998; Weber et al., 2002; Lee et al., 2012; Montanaro and Bryan, 2014; Cahyanto et al., 2016) and were adapted to the present research. The instruments were intensively pre-tested twice with 300 participants each, using a commercial online panel that is representative of Switzerland.^1^ The latent constructs in each case result from the formation of mean value indices from the respective items after prior examination of the factorial validity (main axis analysis with promax rotation) and its reliability (Cronbach’s alpha; Jennrich, 2006). A few items have been discarded due to redundancy. Table 3 illustrates the operationalization of the latent constructs with sample items. The factorial validity and the question items of the survey can be found in detail in Supplementary Table A-1.

**Table 3:** The dimensions of the explanatory model and an associated example item for each model.

Construction	Example item	Number of items	Mean value (*SD*)	Cronbach’s alpha
Risk behaviour (RTB) (*n* = 1,659)	Would you stay in a tent out in the wild, far removed from any town or campsite? (1 = *very unlikely* to 5 = *very likely*)	3	1.91 (1.06)	0.75
Perceived susceptibility (SUS) (*n* = 1,659)	It’s likely that I will be exposed to the coronavirus when travelling at this time. (1 = *do not agree at all* to 5 = *agree entirely*)	4	3.54 (1.10)	0.90
Perceived severity (SEV) (*n* = 1,664)	Getting infected with the coronavirus would have severe consequences for my physical health. (1 = *do not agree at all* to 5 = *agree entirely*)	4	3.27 (1.13)	0.87
Perceived benefits (BEN) (*n* = 1,658)	The protective measures reduce the risk of infection when people travel. (1 = *do not agree at all* to 5 = *agree entirely*)	4	3.78 (0.91)	0.83
Perceived barriers (BAR) (*n* = 1,659)	For me, the effort of applying protective measures when travelling is greater than the benefits. (1 = *do not agree at all* to 5 = *agree entirely*)	4	2.98 (1.13)	0.82
Self-efficacy (SE) (*n* = 1,666)	With my behaviour, I can help to keep infection rates from increasing further during the pandemic. (1 = *does not apply at all* to 5 = *applies entirely*)	4	4.02 (0.90)	0.82
Attitude (N)PI (ATT) (*n* = 1,657)	I find applying the protective measures against the coronavirus when travelling (e.g., wearing masks, quarantining when entering a country, distancing, etc.) to be … (1 = *bad/*etc. to 5 = *good/*etc.)	8	4.27 (0.90)	0.96
Subjective norm (N)PI (SNO) (*n* = 1,644)	Most people who are important to me support the idea of applying protective measures when travelling. (1 = *does not apply at all* to 5 = *applies entirely*)	6	4.15 (0.90)	0.96
Perceived behavioural control (N)PI (PBC) (*n* = 1,654)	It’s easy for me to apply protective measures when travelling. (1 = *does not apply at all* to 5 = *applies entirely*)	4	4.42 (0.67)	0.81
Intention to implement interventions against COVID-19 (INT) (*n* = 1,650)	I firmly intend to apply protective measures on my next trip, even though they are voluntary. (1 = *does not apply at all* to 5 = *applies entirely*)	4	4.04 (1.11)	0.97

SD, standard deviation.

## Results

### Analysis of the Response Rate

The structure of the respondents corresponds to that of the Swiss resident population x§, taking into account the main stratification dimensions of gender, age and language. In Table 4, the socio-demographic characteristics of the respondents are compared with the general Swiss population, as observed in the Swiss Census for 2021 (FSO, 2021). The comparison with the sample shows that the Swiss population is tending to be younger and more highly educated. Age groups and language within our own data follow the official figures. About 39% of the response rate can be considered as satisfying within the Swiss context, given the complexity of our survey instrument (Axhausen and Weis, 2010). However, questionnaire return losses amongst a certain participant group are a problem in cross-sectional studies, as non-response behaviour may be correlated with demographics and the surveyed behaviour. In our case reweighting of the date is not necessary and is limited, as it can be used only for known demographic information and not for information that might be related to norms, milieus, or social status. Thus, given the relatively small deviations, reweighting of the data is unnecessary given the fact that weighting might distort other characteristics.

**Table 4:** Analysis of the response rate differentiated by language and gender, age groups, and educational level.

		Sample [%]	Swiss census [%]
Language	Sex		
German	Male	33	36
	Female	35	36
French	Male	12	12
	Female	15	21
Italian	Male	2	2
	Female	3	2
Age Groups (years)			
	18–30	11	19
	31–55	38	44
	56–65	21	16
	65+	28	21
Education	Compulsory and vocational training	47	46
	Grammar school	8	9
	Higher education	20	15
	Tertiary education	25	30

Own data compared with FSO census for 2021.

### Hypothesis Testing

To test hypotheses H1.1a and H1.1b of conceptually independent predictors and common method bias, all nine predictors were entered into a multiple linear regression model. The Variance Inflation Factor (VIF) for the construct of risk-taking behaviour (VIF = 1.137), perceived susceptibility (VIF = 1.413), perceived severity (VIF = 1.491), perceived benefits (VIF = 1.334), perceived barriers (VIF = 1.247), self-efficacy (VIF = 1.564), attitude towards the behaviour of implementing (N)PIs whilst travelling (VIF = 2.276), subjective norm (VIF = 1.846), and perceived behavioural control (VIF = 1.567) were all below the commonly accepted critical value of 10 (e.g., Belsley et al., 1980).

In Table 5, the intercorrelation matrix of all influencing variables and the dependent variable is presented. We can confirm that the combination of nine explanatory factors into one model is based on the perspective of avoiding possible multicollinearity (no correlation is above 0.65), providing evidence for H1.1a.

**Table 5:** Intercorrelation matrix.

	RTB	SUS	SEV	BEN	BAR	SE	ATT	SNO	PBC	INT
RTB	1									
SUS	−0.206^***^	1								
SEV	−0.308^***^	0.458^***^	1							
BEN	−0.099^***^	0.024	0.160^***^	1						
BAR	0.071^**^	0.011	−0.065^**^	−0.302^***^	1					
SE	−0.150^***^	0.251^***^	0.345^***^	0.375^***^	−0.219^***^	1				
ATT	−0.256^***^	0.387^***^	0.414^***^	0.378^***^	−0.354^***^	0.508^***^	1			
SNO	−0.190^***^	0.294^***^	0.341^***^	0.328^***^	−0.280^***^	0.448^***^	0.617^***^	1		
PBC	−0.144^***^	0.213^***^	0.202^***^	0.353^***^	−0.282^***^	0.432^***^	0.499^***^	0.502^***^	1	
INT	−0.262^***^	0.338^***^	0.401^***^	0.270^***^	−0.293^***^	0.454^***^	0.598^***^	0.520^***^	0.463^***^	1

RTB, Risk-taking behaviour; SUS, Perceived susceptibility; SEV, Perceived severity; BEN, Perceived benefits; BAR, Perceived barriers; SE, Self-efficacy; ATT, Attitude towards the behaviour; SNO, Subjective norm; PBC, Perceived behavioural control; and INT, Intention to adhere to (N)PIs whilst travelling.

** *p* < 0.01.

*** *p* < 0.001, two-tailed (pairwise deletion of cases).

Furthermore, common method variance with Harman’s single factor test was tested. We conducted a principal factor analysis and defined ex ante that only one factor should be extracted. This single factor had an eigenvalue of 15.821 and explained 33.949% of common item variance, which is far less than the threshold of 50%. We can therefore be confident that CMB in this case had been avoided, providing evidence for H1.1b.

**Figure 2:**
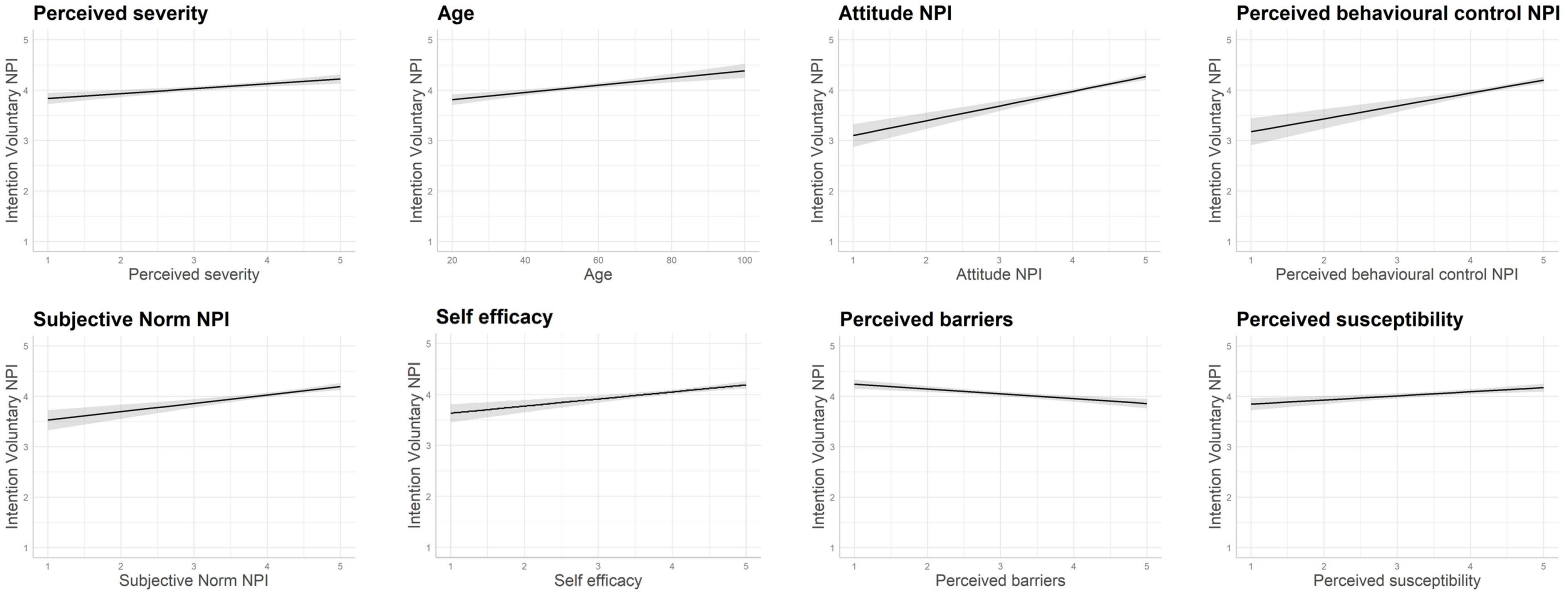
Effect plots to illustrate the effect size for Model 4 in descending order from left to right only for significant effects (absolute value).

**Figure 3:**
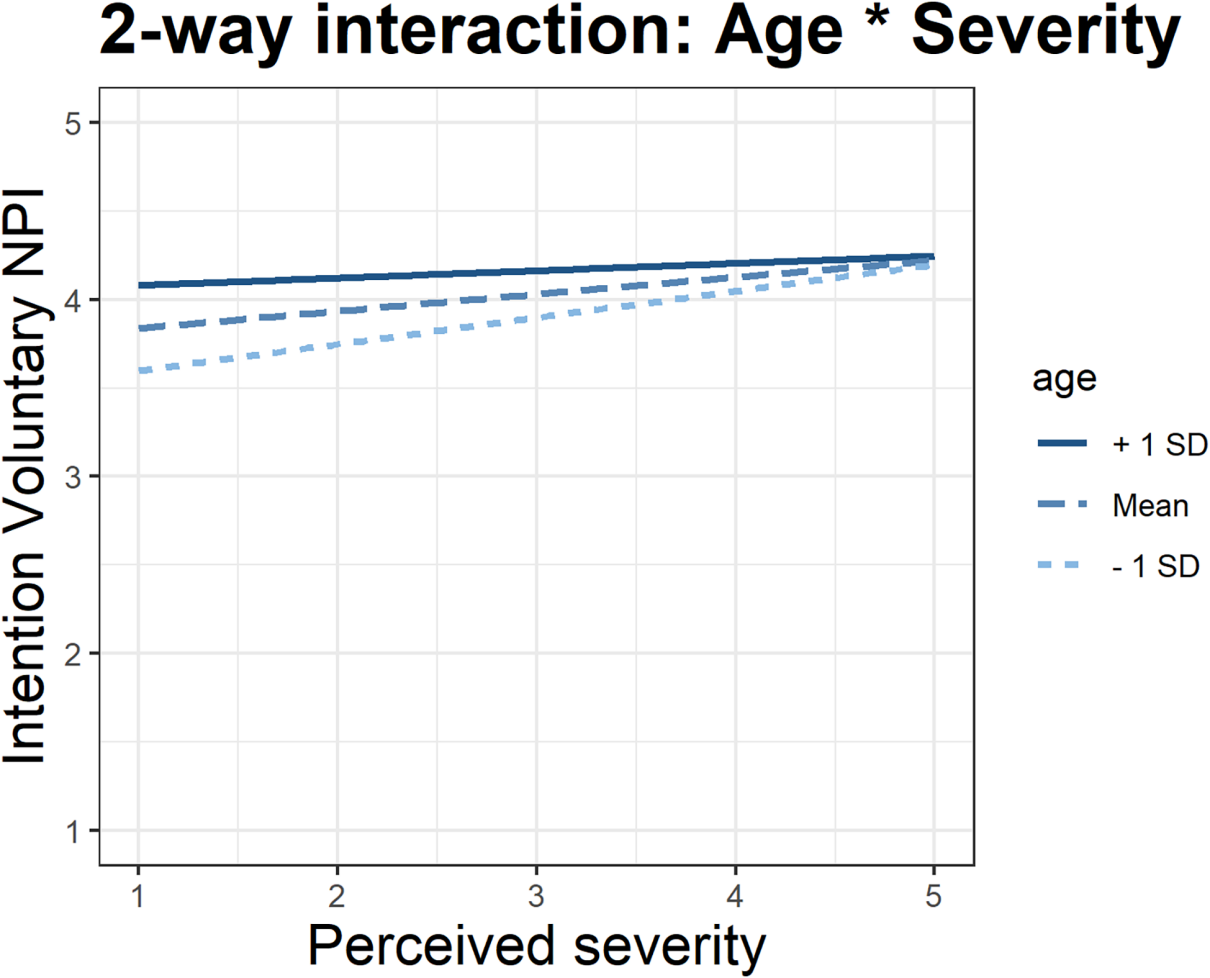
Two-fold interaction effect between age and severity.

Table 6 presents the modelling results using a block-wise comparison of four different model stages. With regard to H1.2, by comparing the corrected R-squared, it can be confirmed that the combined model has greater explanatory power overall. The increase in explanatory power for each model stage is significant based on the ANOVA regression table (see Table 7). This result shows that the best-fit model is number 4, including the interaction term, which leads to a better discussion and assessment of the effect sizes and direction with regard to the causation of the model (*ceteris paribus*). Based on Model 4, H2.1 has to be rejected, namely that greater general risk-taking behaviour in tourism influences the intention to implement interventions against COVID-19. With regard to HBM, H2.2, H2.3, H2.5, and H2.6 can be accepted. Susceptibility, severity, barriers, and self-efficacy influence the intention to implement COVID-19 measures. H2.4 cannot be confirmed, as the perceived benefits are not significant. All constructs of TPB are significant with the postulated direction, confirming H2.7, H2.8, and H2.9. The ranking of significant factors according to their size of effect can be compared by using the standardised coefficients (Beta), together with effect plots for the sake of illustration (see Figure 2). With regard to H3, we find that severity is the leading factor of the model, followed by age (confirms H5) and attitude. Thus, we cannot confirm H3, even though susceptibility is significant, though it plays only a minor role with regard to its effect size. H4 is rejected because, in the final model, gender makes no difference if other variables are controlled for (in comparison to model 1). Since severity and age seem to be the leading factors of the models, following Das and Tiwari (2021, p. 237), we include an interaction effect. Figure 3 shows the effect of severity for mean age, mean age minus one SD, and mean age plus one SD. The interaction effects show that the slope for the younger group is steeper, indicating that the assessment of severity is leading to greater variations in implementing interventions, whereas the effect of severity has little effect on the intention to implement COVID-19 protective measures whilst travelling. Elderly people appear to have a general high intention to adhere to COVID-19 protective measures whilst travelling in general. Their evaluation of perceived severity does play a minor role for increasing their acceptance of protective measures in comparison to younger tourists. H6 is rejected. We found a significant interaction effect, however, the effect was not in the postulated direction.

**Table 6:** Modelling results (with bloc-wise comparison of different model stages).

Effect	*Model 1*			*Model 2*			*Model 3*			*Model 4*		
	*b*	*Beta*	*p*	*b*	*Beta*	*p*	*b*	*Beta*	*p*	*b*	*Beta*	*p*
Intercept	3.442		<0.001	1.690		<0.001	−0.053		0.804	−0.620		0.035
Gender (0 = female)	−0.137	−0.062	0.011	−0.049	−0.022	0.291	−0.006	−0.003	0.880	−0.006	−0.003	0.895
Age (years)	0.018	0.263	<0.001	0.008	0.126	<0.001	0.007	0.103	<0.001	0.018	0.264	<0.001
Risk behaviour (DOSPERT)	−0.155	−0.147	<0.001	−0.068	−0.064	0.005	−0.043	−0.041	0.055	−0.039	−0.037	0.082
Perceived suspectibility (HBM)				0.184	0.179	<0.001	0.080	0.078	<0.001	0.082	0.080	<0.001
Perceived severity (HBM)				0.137	0.138	<0.001	0.093	0.094	<0.001	0.271	0.273	<0.001
Perceived benefits (HBM)				0.073	0.059	0.010	−0.038	−0.030	0.160	−0.038	−0.030	0.158
Perceived barriers (HBM)				−0.204	−0.204	<0.001	−0.100	−0.100	<0.001	−0.096	−0.096	<0.001
Self-efficacy (HBM)				0.318	0.257	<0.001	0.141	0.114	<0.001	0.139	0.112	<0.001
Attitude (N)PI (TPB)							0.297	0.242	<0.001	0.291	0.237	<0.001
Subjective norm (N)PI (TPB)							0.167	0.134	<0.001	0.167	0.134	<0.001
Perceived behavioural control (N)PI (TPB)							0.254	0.150	<0.001	0.256	0.152	<0.001
Age * Perceived Severity (HBM)										−0.003	−0.284	0.005
*R* ^2^_cor._	0.125			0.369			0.464			0.466		
Model comparison (*F*-Test)	*F*(3, 1560) = 75.278, *p* = <0.001			*F*(8, 1555) = 115344, *p* = <0.001			*F*(11, 1552) = 123.969, *p* = <0.001			*F*(12, 1551) = 114.789, *p* < 0.001		
*n* (listwise based on Model 4)	1,564			1,564			1,564			1,564		

b, coefficient estimate; Beta, standardised coefficient estimate; *p*, value of p; *R*^2^_cor._, corrected *R*^2^. Listwise deletion of cases based on Model 4.

**Table 7:** ANOVA regression table.

Model	*R*-squared	Corrected *R*-squared	Changes in *R*-squared	Change in *F*-Statistik	df1	df2	Change in significance
1	0.126	0.125	0.126	75.278	3	1560	<0.001
2	0.372	0.369	0.246	121.883	5	1555	<0.001
3	0.468	0.464	0.095	92.609	3	1552	<0.001
4	0.470	0.466	0.003	7.817	1	1551	<0.01

*n* = 1,564 (listwise deletion of cases).

## Discussion

### Policy Implications

The interventions’ effectiveness is largely dependent on people’s acceptance of them and their intention to implement them correctly. With regard to intervention design, we follow two-step-approach of Aiken (2011, p. 612), which suggests that “[t]he first stage involves the development and evaluation of a psychosocial model of the putative determinants of a particular health behaviour. […] The second stage involves translation of the psychosocial model into a multicomponent intervention to encourage behaviour adoption.” We argue that identifying important influencing factors may guide practical debates on pointers to interventions and safety measures in order to allocate them more effectively to travellers’ decision-making.

**Figure 4:**
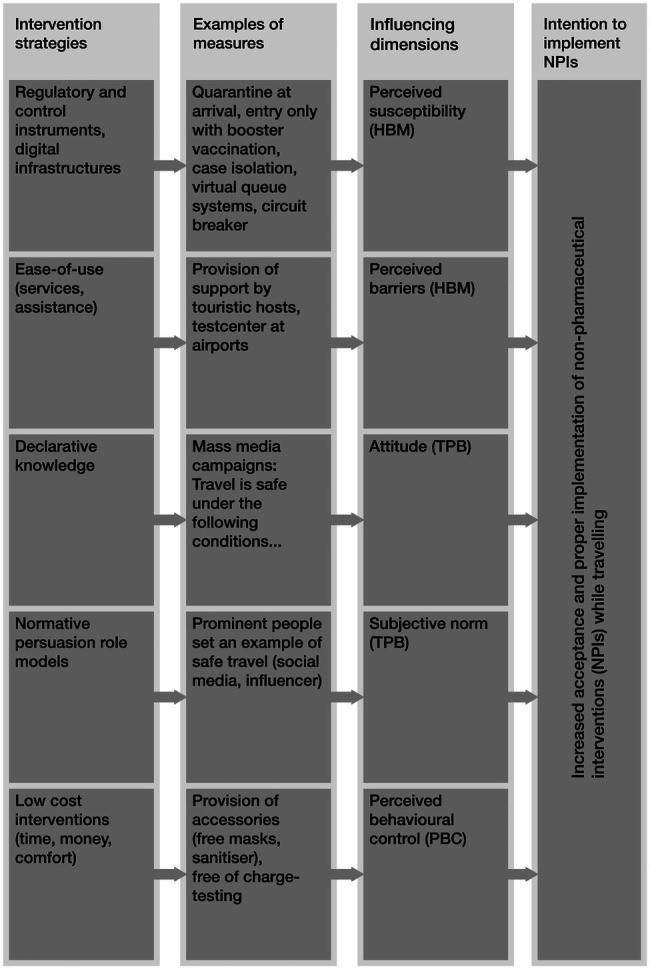
Presentation of exemplary measures for addressing social-psychological influencing dimensions.

According to two-step approach Aiken (2011), a workshop with tourism experts was organised by the project team. Oberholzer et al. (2022) documents how the tourism practitioners found our model useful in assessing COVID-19 measures for the Swiss population. In the workshop, our modelling results served as the backbone layer for a discussion of the effectiveness of various safety measures for Swiss tourism destination managers. The intervention strategies were assigned to the socio-psychological factors based on the explanatory model (Figure 1) and the empirically confirmed influencing dimensions, as well as the strength of their effects on the intention to implement NPIs in the domain of tourism (Table 6; Figure 2; see Ohnmacht et al., 2017, for the case of energy research). These discussions have resulted in Figure 4, which shows a cascade where first intervention strategies are formulated, and then corresponding protective measures are linked to the influencing dimensions.

We suggest that interventions that address positive attitudes towards protective measures (through declarative knowledge within mass media campaigns) and decrease the perceived severity (in fact, vaccinations) should be prioritised, followed by measures addressing perceived behavioural control, subjective norms, and barriers. For example, by way of low-cost interventions for tourists (less time, less money, and more comfort), such as the free provision of accessories (free mask and sanitizers) or free testing (at cable cars), can increase the perceived behavioural control and lower the perceived barriers and thus increase the acceptance of this protective measure. Based on the intervention strategy of normative persuasion role models, prominent tourism ambassadors set an example of safe travel by promoting (N)PIs. This in turn increases the subjective norm, which is translated into increased acceptance of (N)PIs.

### Theoretical Implications and Future Research

The findings of this research project contribute to the existing literature on TPB and HBM with regard to tourists’ perceptions of risk during pandemics and their acceptance of interventions, supporting the results of Bae and Chang (2020), Das and Tiwari (2021), Lee et al. (2012), and Liu et al. (2021). Contrary to Cahyanto et al. (2016) and Huang et al. (2020), perceived severity was significant in our study.

According to this stream of literature, this paper has provided numerous scientific contributions. Firstly, a combined theory-based explanatory model has been developed based on data that are representative of the Swiss population. Secondly, an empirical approach to measuring and predicting tourist intentions in order to implement interventions against COVID-19 whilst travelling has been proved applicable. Thirdly, the combined theory-based model and its empirical results have proved useful in assessing interventions and safety measures based on an expert workshop (Oberholzer et al., 2022).

The study has the limitation that we have applied a simple modelling approach. Our decision to use (simple) multiple linear regression instead of e.g., structural equation models (SEM) is based upon the following reasons. SEM is particularly appropriate when multiple and simultaneous relationships between several independent and dependent variables are to be tested. This is applied in the case of hypotheses that the influence of an independent variable on the dependent variable is mediated through a third variable, or when moderation effects are invoked (e.g., Nunkoo and Ramkissoon, 2012). The goal of our approach was not to detect complex relationships amongst several independent and dependent variables. The aim of the present paper is, to extend the TPB with other relevant predictors to explain the intention to accept measures and interventions in a pandemic. A simple regression approach has the advantage that it is suitable for use by tourism practitioners to derive and evaluate measures for safe travel based on the significant influencing factors (Oberholzer et al., 2022).

This limitation leads to implications for future lines of research. Firstly, more complex modelling approaches should be tested on our data. Secondly, other possible confounding and intervening variables should be considered, such as cue to actions (experience with a former COVID-19 infection) or lifestyle groups, social milieus, or identifiers for corona sceptic groups.

## Data Availability Statement

The datasets presented in this study can be found in online repositories. The names of the repository/repositories and accession number(s) can be found at: https://doi.org/10.5281/zenodo.5941549 Zenodo.

## Ethics Statement

Ethical review and approval was not required for the study on human participants in accordance with the local legislation and institutional requirements. The patients/participants provided their written informed consent to participate in this study.

## Author Contributions

TO and AH contributed to conception and design of the study. AH organised the database and performed the statistical analysis. TO, AH and VT wrote sections of the manuscript. All authors contributed to the article and approved the submitted version.

## Funding

This research was supported by the Swiss National Science Foundation (SNSF) within the framework of the National Research Programme “COVID-19” (NRP 78) Grant-No 4078P0_198336. Open access funding was provided by Lucerne University of Applied Sciences and Arts.

## Conflict of Interest

The authors declare that the research was conducted in the absence of any commercial or financial relationships that could be construed as a potential conflict of interest.

## Publisher’s Note

All claims expressed in this article are solely those of the authors and do not necessarily represent those of their affiliated organizations, or those of the publisher, the editors and the reviewers. Any product that may be evaluated in this article, or claim that may be made by its manufacturer, is not guaranteed or endorsed by the publisher.

## Footnotes

^1^ www.respondi.com
