# Supplementary material for: Pointers to Interventions for Promoting COVID-19 Protective Measures in Tourism: A Modelling Approach Using Domain-Specific Risk-Taking Scale, Theory of Planned Behaviour, and Health Belief Model
Source: Front Psychol. 2022 Jun 29;13:940090. doi: 10.3389/fpsyg.2022.940090 (PMC9277178; doi:10.3389/fpsyg.2022.940090)
Supplement: Supplementary file 1 [file Table_1.docx]

Table A-1. Principal axis factor-analysis (Rotation: Promax (κ = 4) with Kaiser-Normalization)

| **Item** | **Question** | **RTB** | **SUS** | **SEV** | **BEN** | **BAR** | **SE** | **ATT** | **SNO** | **PBC** | **INT** | ***n*** |
| --- | --- | --- | --- | --- | --- | --- | --- | --- | --- | --- | --- | --- |
| rtb_1 | Would you stay in a tent out in the wild, far removed from any town or campsite? | **-.634** | -.018 | -.014 | -.002 | .043 | .005 | -.031 | -.001 | -.001 | -.013 | 1,476 |
| rtb_2 | Would you join a whitewater rafting tour in fast-flowing rivers in the spring? | **-.789** | -.010 | -.008 | -.007 | -.039 | .024 | -.014 | -.002 | .008 | .039 | 1,476 |
| rtb_3 | Would you do risky sports (e.g. rock climbing, skydiving, etc.) regularly? | **-.771** | .022 | .025 | -.004 | .016 | .024 | .012 | -.015 | -.019 | -.021 | 1,476 |
| sus_1 | It's likely that I will be exposed to the coronavirus when travelling at this time. | -.070 | **.693** | .007 | .008 | -.039 | -.036 | .026 | .017 | .033 | -.041 | 1,476 |
| sus_3 | There is currently a high risk of infection from the coronavirus when travelling. | .041 | **.879** | -.001 | .008 | -.007 | .015 | -.030 | -.004 | -.008 | .047 | 1,476 |
| sus_4 | There is currently a high risk of passing on the coronavirus when travelling. | .015 | **.835** | .027 | -.048 | .036 | .054 | .009 | -.011 | -.030 | -.003 | 1,476 |
| sus_5 | There is currently a high risk of coming into contact with the coronavirus when travelling. | .022 | **.950** | -.026 | .016 | .003 | -.005 | -.014 | -.010 | .005 | .001 | 1,476 |
| sev_1 | Getting infected with the coronavirus would have severe consequences for my social life (friends, club, sport). | -.004 | .042 | **.622** | .016 | -.035 | -.011 | .057 | .027 | .012 | -.023 | 1,476 |
| sev_2 | Getting infected with the coronavirus would have severe consequences for my physical health. | .006 | .041 | **.763** | -.009 | .017 | .006 | -.013 | .050 | .014 | .052 | 1,476 |
| sev_3 | Getting infected with the coronavirus would have severe consequences for my mental well-being. | -.007 | -.045 | **.911** | -.028 | -.030 | -.001 | .015 | -.017 | .003 | -.010 | 1,476 |
| sev_4 | Getting infected with the coronavirus would have severe consequences for my mental ability to perform. | .002 | -.010 | **.827** | .009 | .034 | .008 | -.049 | -.033 | -.030 | .011 | 1,476 |
| ben_3 | The protective measures effectively contain the coronavirus when people travel. | .059 | -.014 | .074 | **.822** | .010 | .038 | -.008 | -.050 | .003 | -.030 | 1,476 |
| ben_4 | The protective measures reduce the risk of infection when people travel. | -.045 | .050 | .009 | **.842** | .008 | -.048 | .049 | .013 | -.016 | -.045 | 1,476 |
| ben_5 | The protective measures make me feel safe when I travel. | .028 | -.057 | -.064 | **.739** | .026 | .045 | -.049 | .010 | -.048 | .042 | 1,476 |
| ben_6 | By applying protective measures while travelling, I am behaving responsibly. | -.043 | .010 | -.051 | **.471** | -.058 | -.002 | .018 | .049 | .103 | .111 | 1,476 |
| bar_1 | For me, the costs (time, comfort, money) of applying protective measures when travelling are greater than the benefits. | .045 | -.058 | -.020 | -.050 | **-.764** | .065 | -.034 | .023 | -.011 | .048 | 1,476 |
| bar_2 | For me, the effort of applying protective measures when travelling is greater than the benefits. | .054 | -.025 | -.038 | -.058 | **-.792** | .059 | -.009 | -.004 | -.031 | .057 | 1,476 |
| bar_3 | The protection measures are disturbing when travelling. | -.054 | .054 | .025 | .056 | **-.658** | -.067 | .032 | -.041 | .002 | -.054 | 1,476 |
| bar_4 | The protection measures prevent pleasant travelling. | -.033 | .047 | .048 | .048 | **-.700** | -.044 | -.005 | .004 | .050 | -.067 | 1,476 |
| se_1 | With my behaviour, I can help to keep infection rates from increasing further during the pandemic. | .000 | .031 | -.031 | -.011 | -.023 | **.657** | .085 | .012 | .060 | .009 | 1,476 |
| se_2 | I can contribute to ending the pandemic soon. | .031 | -.010 | .073 | -.015 | .011 | **.770** | .018 | -.048 | .032 | -.058 | 1,476 |
| se_3 | I can help protect society from the coronavirus. | -.056 | .012 | -.044 | .015 | .005 | **.844** | -.040 | -.003 | -.087 | .014 | 1,476 |
| se_4 | Risk groups are best protected if I apply the measures. | -.019 | -.004 | .018 | .058 | -.034 | **.579** | .027 | .100 | -.001 | .015 | 1,476 |
| att_npi_1 | I find applying the coronavirus protective measures when travelling to be … bad - good | .035 | .010 | -.039 | -.011 | .040 | .062 | **.813** | -.018 | -.003 | -.047 | 1,476 |
| att_npi_2 | … useless - useful | -.007 | -.010 | -.007 | .048 | -.021 | .014 | **.871** | .028 | -.001 | -.046 | 1,476 |
| att_npi_3 | … not desirable - desirable | .005 | .018 | .007 | -.058 | .036 | .039 | **.702** | .010 | -.031 | .094 | 1,476 |
| att_npi_4 | … inappropriate - appropriate | -.033 | .020 | -.007 | .007 | .003 | -.064 | **.946** | .004 | .008 | -.020 | 1,476 |
| att_npi_6 | … unimportant - important | -.011 | .021 | .002 | -.008 | -.039 | -.001 | **.884** | .030 | .005 | .025 | 1,476 |
| att_npi_7 | … not worthwhile - worthwhile | .054 | -.063 | .029 | .017 | .035 | .073 | **.809** | -.035 | -.017 | -.033 | 1,476 |
| att_npi_8 | … unnecessary - necessary | -.013 | .005 | -.004 | .000 | -.041 | -.057 | **.920** | .035 | -.017 | .053 | 1,476 |
| att_npi_9 | … meaningless - meaningful | .004 | -.012 | .018 | -.006 | .010 | -.022 | **.922** | -.009 | -.008 | .008 | 1,476 |
| sno_npi_2 | Most people who are important to me are in favor of applying protective measures when travelling. | -.024 | .043 | .014 | -.026 | .024 | -.044 | .097 | **.825** | -.003 | .010 | 1,476 |
| sno_npi_3 | Most people who are important to me think that applying protective measures when travelling is a good idea. | -.013 | .032 | .006 | .006 | .030 | -.037 | .071 | **.872** | -.015 | -.010 | 1,476 |
| sno_npi_4 | Most people who are important to me think I should apply protective measures when travelling. | .016 | -.019 | -.002 | .000 | -.011 | -.009 | -.016 | **.913** | -.006 | .006 | 1,476 |
| sno_npi_5 | Most people who are important to me generally recommend applying protective measures when travelling. | -.009 | -.012 | -.026 | .009 | .004 | -.010 | .000 | **.931** | -.009 | .019 | 1,476 |
| sno_npi_6 | Most people who are important to me support me in applying protective measures when travelling. | .025 | -.017 | .006 | -.004 | -.012 | .049 | -.007 | **.871** | .036 | -.051 | 1,476 |
| sno_npi_7 | Most people who are important to me encourage me to apply protective measures when travelling. | .016 | -.023 | .011 | .012 | -.015 | .051 | -.063 | **.891** | -.020 | .003 | 1,476 |
| pbc_npi_1 | I am confident that I will apply protective measures when travelling. | -.002 | .006 | .008 | .021 | .006 | .025 | .143 | .067 | **.543** | .081 | 1,476 |
| pbc_npi_2 | I know how to apply protective measures correctly when travelling. | .045 | .015 | .017 | .023 | .002 | -.021 | -.056 | -.003 | **.789** | -.053 | 1,476 |
| pbc_npi_3 | I am able to apply protective measures correctly when travelling. | -.032 | -.003 | -.019 | -.016 | -.054 | -.056 | -.022 | -.018 | **.961** | -.023 | 1,476 |
| pbc_npi_4 | It's easy for me to apply protective measures when travelling. | .008 | -.025 | -.012 | -.039 | .128 | .125 | .022 | -.006 | **.489** | .072 | 1,476 |
| int_v_npi_1 | I will probably apply protective measures on my next trip, even though they are voluntary. | -.019 | .003 | -.005 | -.008 | .006 | .002 | .006 | -.025 | -.004 | **.919** | 1,476 |
| int_v_npi_2 | I will definitely apply protective measures on my next trip, even though they are voluntary. | .020 | .003 | .011 | .002 | .007 | -.001 | -.006 | -.008 | -.014 | **.950** | 1,476 |
| int_v_npi_3 | I firmly intend to apply protective measures on my next trip, even though they are voluntary. | .033 | .009 | .016 | .022 | .004 | -.013 | .006 | .013 | -.002 | **.907** | 1,476 |
| int_v_npi_4 | I am willing to apply protective measures on my next trip, even though they are voluntary. | -.035 | -.014 | .001 | .015 | -.010 | -.010 | .039 | .014 | -.006 | **.938** | 1,476 |

*Note*. N = 1,683. Missing cases were deleted listwise (n = 1,476), Kaiser-Meyer-Olkin = 0.948. Bartlett-Test (Sphericity): X^2^ (990, N = 1476) = 53668.415, p < .001.

The ten factors together explain (in total) 67.517% of variance
